# A Self-Polymerizing Mesh of Nano-Tethers for the Mechanical Constraint of Degraded Intervertebral Discs—A Review of 25 Years of Pre-Clinical and Early Clinical Research

**DOI:** 10.3390/bioengineering11060535

**Published:** 2024-05-24

**Authors:** Thomas Hedman, Adam Rogers, Douglas Beall

**Affiliations:** 1 F. Joseph Halcomb III, M.D. Department of Biomedical Engineering, University of Kentucky, Lexington, KY 40513, USA; 2 Spinal Simplicity LLC, Overland Park, KS 66211, USA; 3 Comprehensive Specialty Care, Edmond, OK 73034, USA

**Keywords:** medical innovation, disc degradation, low back pain, lumbar spine, polymers, degenerative disc disease, discogenic back pain

## Abstract

Genipin polymers are self-forming tensile-load-carrying oligomers, derived from the gardenia fruit, that covalently bond to amines on collagen. The potential therapeutic mechanical benefits of a non-discrete in situ forming mesh of genipin oligomers for degraded spinal discs were first conceived in 1998. Over more than two decades, numerous studies have demonstrated the immediate mechanical effects of this injectable, intra-annular polymeric mesh including an early demonstration of an effect on clinical outcomes for chronic or recurrent discogenic low back pain. This literature review focused on articles investigating mechanical effects in cadaveric animal and human spinal discs, biochemical mechanism of action studies, articles describing the role of mechanical degradation in the pathogenesis of degenerative disc disease, initial clinical outcomes and articles describing current discogenic low back pain treatment algorithms. On the basis of these results, clinical indications that align with the capabilities of this novel injectable polymer-based treatment strategy are discussed. It is intended that this review of a novel nano-scale material-based solution for mechanical deficiencies in biologically limited tissues may provide a helpful example for other innovations in spinal diseases and similarly challenging musculoskeletal disorders.

## 1. Introduction

Innovation in medical technologies necessarily involves multi-layered research strategies. The more unconventional the potential medical product is, the more valid questions will arise and the more data will be required to convincingly establish its merits, limitations, and appropriate use. An unavoidable companion on this innovation journey is the ever-present educational obligation to alert all potential stakeholders of the new technology’s capabilities and mechanisms of action.

The clinical need that led to innovation in this case was the highly prevalent and poorly addressed disorder involving recurrent and chronic low back pain (LBP) stemming from progressively degenerating lumbar intervertebral discs. This malady is often referred to as discogenic low back pain. The progressive loss of a lumbar disc’s mechanical sufficiency is universal to some degree, due to the high stresses in these load-bearing tissues combined with the harsh biological environment and limited healing potential in the largely avascular disc tissues [1,2,3,4]. Under these conditions, microstructural damage accumulates, resulting in increasingly overstressed and vulnerable collagenous tissues supporting rarely decreasing upper body weight while being involved in an array of physiological motions.

The primary load-supporting and motion-constraining component of the lumbar disc is the annulus fibrosus [4]. The annulus sheets (lamellae) of collagen fibers support high levels of primarily tensile stresses. Early microstructural damage to the disc tissues including the loss of water-imbibing molecules (proteoglycans) leads to joint instability and increased solid phase load support in the annulus fibrosus [3,5,6]. Increased solid phase stresses combined with an accumulation of tissue degradation due to limited tissue repair can result in the disc annulus becoming increasingly unable to withstand the stresses and strains of physiological loading, leading to a degenerative cascade of additional tissue damage, increased tissue stresses and strains, and the increased vulnerability of the tissue to stress overload [1,7]. The increasing mechanical insufficiency of degenerating discs can also lead to overloading and degenerative changes in the adjacent joints and surrounding tissues [8,9,10]. While degenerative characteristics associated with painful discs can be more readily observed using imaging in later stage disease (e.g., the visualization of diffuse annular tearing), early- to mid-stage discogenic low back pain is generally non-specific (or idiopathic, estimates of around 85% [11] or 90% [12]), meaning that there is usually no confirmed pathoanatomical cause for the discogenic pain [12]. This is not to say that painful lumbar discs seen in association with early- to mid-stage low back pain are assumed to be degeneration-free and mechanically sufficient, as it is known from anatomic studies that lumbar discs undergo age-related changes from birth [1,3,5,13]. Joint instability [14], excessive strain in peripheral disc tissues, and aberrant disc bulging may be contributory to discogenic pain, but they are not measurably discriminatory at this stage of disc degradation. At the same time, it is important to understand that the majority of low back pain patients describe a gradual onset of symptoms [13], and that more than two-thirds of recent onset cases and four-fifths of non-recent onset cases are either recurrent (episodic and lasting more than a year) or chronic [15,16].

Intervertebral disc degeneration (IDD)-associated low back pain is the second most common painful condition with reported point prevalence rates between 12% and 45% [17,18,19,20] resulting in lost work time [17,21] and 10–12% of the population being disabled by chronic LBP [3,17,18,19]. As stated by Maher et al. [12], “Low back pain is the leading chronic health problem forcing older workers to retire prematurely and forcing more people out of the workplace than heart disease, diabetes, hypertension, neoplasm, respiratory disease, and asthma combined”. At present, treatment options for this condition range from pain masking strategies such as over-the-counter drugs, opioid and other non-opioid pain management, neuromodulation, nerve ablation, physical therapy, and steroid injections. Other strategies attempt to eliminate neural compression or improve the load support in joint tissues through methods including nucleus augmentation, stem cell treatments, discectomy, fusion, and disc prostheses.

**Figure 1:**
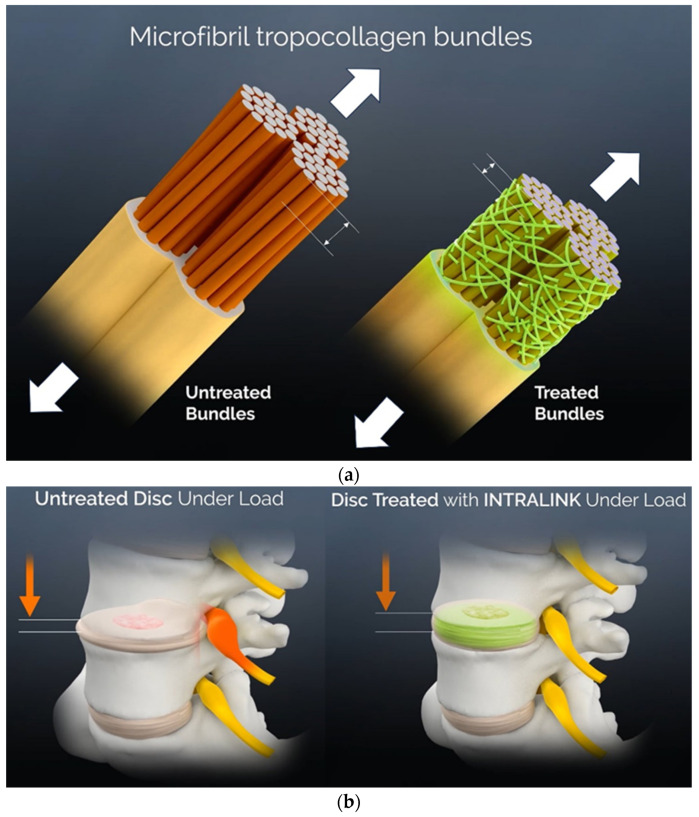
(**a**) A schematic showing the constrained deformation of collagen microfibrils under a tensile load due to the attached, load-sharing mesh of genipin polymers. Large white arrows represent the tensile forces on the collagen fibrils and the small arrows indicate the amount of fibril stretch of the genipin mesh-augmented fibril vs. the untreated fibril under the same force. (**b**) A schematic showing how the constraint of the tensile elongation of annulus collagen microfibrils by genipin mesh translates to a reduction in disc compression and the outward bulging of the disc under compressive loads. The arrows represent the direction but not the location of the applied compressive force and the white lines represent the amount of deformation in the genipin mesh-augmented disc vs. the untreated disc under the same compressive force. This increased outward bulge under a load could impact the strain of imbedded nociceptive nerves [1] or radicular symptoms (an exaggerated depiction is shown). The reduced tensile elongation of annulus fibers by load-sharing mesh can also be expected to decrease joint instability [4,14].

The load-carrying, polymeric intervention reviewed here relies on image-guided injections of self-forming genipin oligomers (monomers of varying lengths [22]) into the degraded annulus fibrosus. Genipin is an organic molecule, derived from gardenia fruit, that can bond at the terminal ends of the oligomer to amines on proteins [22,23,24,25]. The therapeutic benefit of the device is derived from the inherent mechanical properties of a non-contiguous mesh of many genipin polymers that mechanically constrain the degraded disc fibrous collagen matrix, reducing aberrant deformations while increasing the load-carrying capability of the disc (Figure 1a), thereby increasing the mechanical stability of the intervertebral amphiarthrodial joint (Figure 1b). Due to its mechanical binding functionality, the injectable genipin mesh is referred to here as “nano-tethers” and has been classified as a medical device in multiple jurisdictions. Considering the causal link between disc mechanical insufficiency, joint instability and the presence of discogenic pain [3,8,14], the investigation reviewed here held a primary focus on determining the mechanical capabilities of the injectable intra-annular polymeric mesh.

## 2. Methods

Articles relevant to the topic were obtained from searches in the Embase, PubMed, Medline, and Cochrane Library databases. Article searches excluded papers pertaining to treatments associated with sclera or dentin tissues, the use of genipin in hydrogel or other biomaterial tissue repair constructs.

## 3. Results

The articles’ results and discussions of their roles and contributions in the development and testing of this novel materials-based technology were divided into five categories somewhat matching the chronology of the research and development journey: the examination of the role of mechanical property degradation in the pathogenesis of IDD, a reaction kinetics review, a mechanical effects review, a clinical studies review, and a review of the current continuum of care for discogenic LBP.

### 3.1. The Role of Mechanical Strength Deterioration in the Pathogenesis of IDD

Intervertebral disc degeneration is a common condition that affects the intervertebral discs in the spine with aging. Genetic factors [26], nutritional deficiencies (most degenerated discs have an impaired nutrient supply [2]), biochemical degradation (with age, there is a depletion of and alteration in the aggrecan molecules in the matrix [27]), and mechanical degradation (loading conditions result in tissue deterioration [28]) are primary factors that cause the discs to irreversibly degrade over time [5]. Due primarily to the nutritional deficiency [2] of this highly stressed [1] avascular tissue, the disc has limited ability for regeneration or repair, allowing tissue damage from physiological loading to accumulate. Disc degradation can eventually lead to a mechanically insufficient disc, for example, a disc with greater amounts of outward bulging under a load, and a less stable intervertebral joint. This instability and loss of tissue strength can lead to increased stress on the degraded disc and a degenerative cascade whereby the disc degrades, load support capability is diminished, disc biology is further impaired, spinal joint motion is less constrained, localized stress concentrations occur, the disc bulges more under a load, and embedded nerve endings, typically in the outer annulus fibrosus region of the disc, become exposed to excessive tissue strain or inflammatory mediators, initiating pain [1,3,14,29]. The clinical incidence of chronic and recurrent LPB has been associated with these and other forms of escalating disc insufficiency [8]. “As the tissue breaks down its mechanical properties degrade to the point where it is unable to withstand the stresses and strains of physiological mechanical loading, resulting in greater outward bulging, tearing and eventual rupture” [30]. While all repair-limited and mechanically degraded discs are not necessarily symptomatic, all persistent low back pain cases have some degree of disc degeneration and the associated mechanical insufficiency in common [13,16].

**Figure 2:**
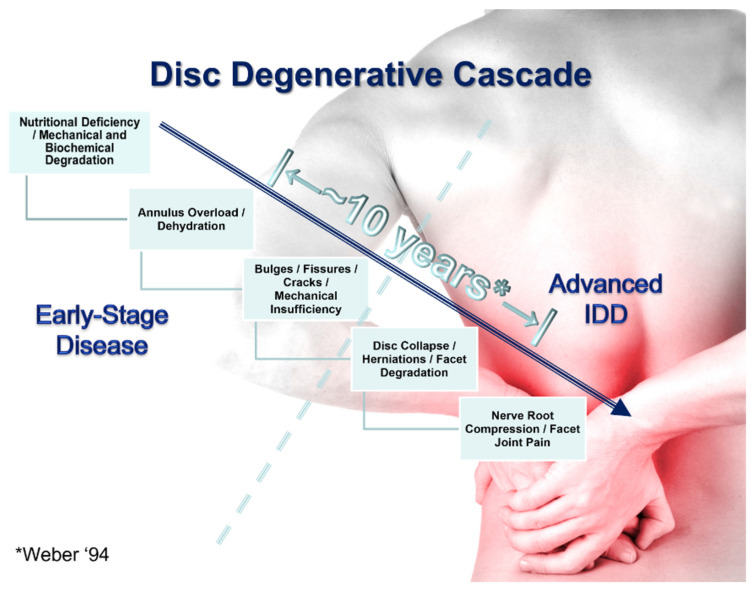
The IDD-associated degenerative cascade leading to increasing stages of debilitating low back and radicular pain. * There is approximately a decade between the first attack of acute low back pain and the onset of radicular symptoms according to Weber [31].

The increasing mechanical insufficiency of degraded discs can lead to further overloading and degenerative changes in the surrounding joints and tissues. If left untreated, or if treatment is exclusively directed at masking or minimizing the associated back pain, it will progress to a more serious condition. Pain masking could make mechanical degradation worse due to an absence of pain avoidance and therefore a continuation of the excessive loading of the damaged tissues. Figure 2 depicts the classical understanding of the lumbar disc degenerative cascade with an indication of the general progression from early-stage recurrent or chronic LBP to more advanced disease. According to Weber [31], the time between the first attack of acute low back pain to the onset of radicular symptoms is typically around a decade. The need for spinal stabilization does not depend upon whether the decades-long degradation of the disc had only precipitated symptoms after a specific loading event (recent onset), or the symptoms had gradually progressed along with the disc degradation for an indefinite period of time (non-recent onset). The mechanically unstable spinal joint and mechanically degraded disc often require mechanical stabilization and support.

### 3.2. The Reaction Kinetics of Genipin in Collagenous Tissues

The process of implanting (or placing) the Intralink genipin-based device into the annulus fibrosus of a disc is accomplished through two separate posterolateral injections to the left and right mid-lateral annulus regions. Beginning immediately prior to injection, the genipin molecules first polymerize and then, after the injection, attach to the collagen matrix in the annulus.

Upon dissolving in an aqueous carrier solution, the genipin will undergo an oxygen radical-induced (typically OH^−^) self-polymerization reaction [22,23,24]. The Intralink product for symptomatic degenerated lumbar discs is contained in two separate vials. One contains genipin powder and the other contains an aqueous buffer. The vials are combined and loaded into a syringe just prior to the image-guided injection procedure. Once the vials are combined, the dissolved genipin begins to self-polymerize.

In 2010 and 2011, Slusarewicz et al. [30,32] demonstrated optimal genipin self-polymerization in a phosphate-buffered aqueous solution with a pH of 9. They further demonstrated that the majority of genipin self-polymerization can be expected to be complete within 10 min. The formation of genipin polymers from genipin molecules is an irreversible reaction, meaning that genipin polymers do not convert back into non-polymerized genipin molecules [24].

After the Intralink liquid-carried polymer device is delivered into the mid-lateral intervertebral disc annulus regions, the genipin continues to undergo an oxygen radical-induced polymerization while dispersing uniformly through the porous extracellular matrix of the annulus fibrosus region. As the newly formed polymers move through the fibrous matrix, they attach to matrix proteins, primarily the collagen on the periphery of disc subfibrils. Polymer attachment is formed by covalent bonds between the ends of the genipin polymers and the available “surface” amine attachment sites in the collagen of the annulus extracellular matrix [22,23,25]. “This reaction begins with an initial nucleophilic attack of the genipin C3 carbon atom from a primary amine group to form an intermediate aldehyde group. Opening of the dihydropyran ring is then followed by attack on the resulting aldehyde group by the secondary amine formed in the first step of the reaction” [23].

Covalent (electron-sharing) bonds are often referred to as “permanent bonds” due to the inherently stable nature of the shared electron pair in the bond. The long-lasting nature of genipin’s covalent bonds to disc collagen has been demonstrated in cadaveric disc tissue repetitive loading studies [33], chronic animal studies [34], and multi-year human clinical studies [35].

### 3.3. A Mechanical Effects Review

The purpose of the disc annulus is to withstand spinal loading by carrying tensile forces in the collagen fibers to constrain the disc and prevent aberrant joint motions and disc deformations (e.g., bulging). Progressive failure of the disc annulus collagen fibers dictates the degree of the overall functionality of the intervertebral disc. The foundational development work for the Intralink product was done to create a device that could rapidly (within minutes or hours) restore the mechanical integrity of these highly loaded collagenous tissues by holding together torn, delaminated, and damaged regions of annulus fibrosus tissues to help the degraded tissues to support physiological loading. With the Intralink mesh of genipin polymers, it was postulated that these degraded fibrous tissues would be held together, or “nano-sutured”, to restore the ability of the tissues to support ongoing physiological loads while reducing or preventing excessive bulging, joint instability, or deleterious high tissue stresses. The studies summarized in this section were intended to investigate the ability of this product to augment or restore intervertebral disc mechanical properties.

Studies involving the use of cadaveric animal and human spine tissues and in vivo animal studies can provide precise measurements of tissue mechanical properties reflecting the mechanisms of action and the magnitude of product effects. Over 20 years of ex vivo experiments demonstrated that the addition of a mesh of self-polymerized genipin attached to the collagen matrix of the intervertebral discs leads to a long list of beneficial changes to the mechanical properties of the spinal disc and the associated intervertebral joint. The magnitudes of some of these mechanical effects are summarized in Table 1.

The addition of genipin polymers to the disc annulus resulted in increased tissue strength (78% circumferential ultimate tensile strength (UTS), *p* = 0.033; 21% axial UTS, *p* = 0.036; 66% yield strength, *p* = 0.012; 36% yield strain, *p* = 0.048; 57% resilience, *p* = 0.019) [32,36,37], a 28% reduction in disc bulge under a load, *p* = 0.001 [38], an increased resistance to tissue tearing and delamination (a delamination peak force per width increase of 70%, *p* < 0.018; the post-tear mean cycles to failure increased from 72 to 3307, *p* = 0.012) [39,40], and to more stable intervertebral joint motion (a range of instability score reductions from 30% to 77%, *p* < 0.05; an axial neutral zone stiffness increase of 91%, *p* < 0.05) [33,41,42,43,44]. Each of these improvements can be expected to address the core mechanical deficiencies associated with IDD and the genesis of discogenic pain and disability. In addition, ex vivo studies have demonstrated that the addition of genipin polymers to the disc annulus fibrosus influences other mechanical constraint-related properties of the disc including moderating fluctuations in intradiscal pressures [45], reducing tensile stresses in the annulus [46], increasing the resistance to degradation from repetitive loading [33], resisting the outward drift of proteoglycans or proteoglycan fragments from the annulus matrix [42] while allowing an increased fluid flow through the matrix [47], providing spinal joint re-stabilization after disc decompression surgery [48], and maintaining the functional integrity of the disc after a penetrating disruption to the annulus [49,50]. Ex vivo spinal joint mechanical stabilization via Intralink injections was observed in a chronic in vivo study of Intralink in a sheep model [34], and, as described below, by kinematic data in an early clinical study [35]. Similarly, the ability of genipin polymers to restore and moderate intradiscal pressures that was demonstrated in ex vivo experiments [45,49] was confirmed in a porcine in vivo experiment [50]. All these mechanical enhancements can contribute to resisting the progression of disc degeneration and the genesis of pain and disability.

**Table 1:** Demonstrated capabilities from preclinical studies of Intralink related to disc degradation and factors related to chronic and recurrent LBP, disability, and degenerative mechanisms.

Pain or Degeneration-Related Mechanical Factor	Effect Size
Resistance to tissue degradation from repetitive loading [33,40]	3-fold increase
Joint stability, neutral zone reductions [33,41,42,43,44] †	Up to 4-fold increase
Annulus mechanical properties such as tensile strength, yield strength, resilience and toughness [32,36,37]	50% or more improvement
Disc bulging under a load [32,38]	38% reduction
Resistance to tear propagation, delamination [39,40]	Up to 70% increase
Tensile stress levels in annulus [47]	3- to 8-fold lower
Annular sealing, disc pressure restoration [49,50]	5- to 7-fold increase

† Also demonstrated in a clinical study with objective kinematic data [35].

As described in detail in the referenced publications, the loss of spinal joint motion constraint is most commonly quantified by neutral zone instability [14]. The neutral zone metric uses joint motion characteristics to evaluate the relative amount of inadequately constrained joint motions. This metric can be thought of as a measure of the insufficient constraint or laxity of the spinal joint. A young, healthy lumbar joint has varying amounts of but continuous resistance to bending (motion constraint) from one extreme of bending to the other (i.e., from full backward bending to full forward bending). An older, degraded spinal joint has a region in the middle of this motion (the neutral zone) where there is very little resistance to bending (i.e., inadequate motion constraint). A poorly constrained joint is associated with excessive tissue strains and subsequent tissue overloading and damage accumulation from normal daily activities. The “instability score”, first described in 2006 [41], is a similar metric based on the neutral zone.

The mechanical improvements resulting from the binding of degraded annulus tissue and the added load support provided by the mesh of genipin polymers occur very rapidly, which is important to providing clinical benefits. This rapid augmentation of the tensile strength of degraded load-supporting tissue was a foundational design objective of this technology, mandating the choice of a non-biological, exclusively materials-based approach rather than a biologic approach for the repair of these biologically challenged tissues.

Other emerging materials-based approaches attempt to remedy the mechanical insufficiency of the disc by providing a different means of load support in the disc. Instead of augmenting the tensile strength of the native collagen matrix, these materials aim to provide direct compressive load support. These alternate approaches use materials, typically hydrogels, with the compressibility and hydrophilicity of native disc tissues. While Intralink adds nano-scale tensile load-carrying polymers to the native tensile load-carrying collagen tissue matrix without displacing any of the disc structures, these bulk materials are intended to fill voids in the degraded disc, either inflating the increasingly fibrous nucleus or the material can be injected as a liquid penetrating into “gaps” in the annulus fibrosus prior to the in situ formation of the solid material [51].

A long-standing challenge to the use of these “nucleus augmentation” or “disc augmentation” materials has been the avoidance of implant extrusion. Even with the more recent introduction of injectable in situ forming polymers, a relatively large diameter disc penetration is required to inject the materials (17 Ga vs. 22 Ga for Intralink). Large diameter needle penetration can lead to both implant material expulsion and a disc penetration injury causing a “drastic alteration” of annulus strain leading to disc degeneration [52,53]. A recent early feasibility study of an injectable in situ forming hydrogel observed the migration or extrusion of implant materials in 15% of these early cases resulting in two serious adverse events [51]. In addition, the void space required by bulking materials is generally not available in early- to mid-stage degenerated discs, essentially removing recurrent discogenic low back pain patients with early- to mid-stage disc degeneration as possible candidates for the treatment. Consequently, compression-resisting disc augmentation materials are indicated for more severely degenerated discs that can accommodate the addition of a bulk material, whereas Intralink is preferably directed toward early- to mid-stage disc degeneration cases. For these reasons, the selection of an injectable, distributed, nano-scale tensile load-carrying implant rather than a bulking agent or other compressive load-carrying, potentially tissue-displacing approach was another foundational design objective for this technology to ensure the preservation of tissue integrity and the normal, primarily tensile load-carrying characteristics of these complex collagenous tissues.

### 3.4. Clinical Studies Review

Beginning in 2016, but interrupted by the response to COVID-19, single-arm, multi-site medical device clinical studies were initiated in Malaysia and Australia. The Malaysian study was completed in 2018 with two-year follow-up on five study participants. The Australian study has enrolled 15 of a maximum of 50 study participants with a 12-month follow-up. Similar clinical protocols including inclusion–exclusion criteria and outcomes were used to facilitate the analysis of combined safety and preliminary efficacy data. National and regional ethics committee approvals were obtained prior to study commencement, and written informed consents were obtained from all study participants prior to enrollment. The initial study results and conclusions described below were reported by Hedman et al. [35].

**Figure 3:**
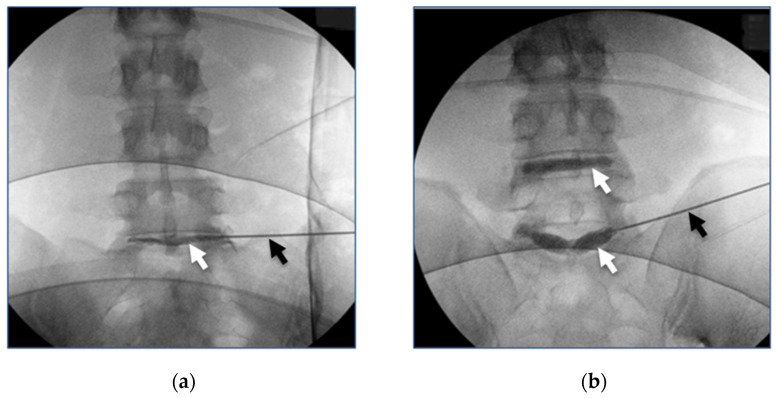
Fluoroscopic images of the Intralink injectate with contrast after bilateral injections in a single-level patient (**a**) and a two-level patient (**b**). The needles (black arrows in (**a**,**b**)) in both patients are seen entering the L5-S1 intervertebral disc and the Intralink mixed with contrast (white arrows in (**a**,**b**)) is easily visualized within the intervertebral discs.

Eligible patients were 18–60 years of age with symptomatic IDD at one or two levels, no imaging or medical history indicating penetrating disc tears, and no indications of other sources of back pain. Results from a total of 20 chronic LBP patients (10 female, 10 male, ages 24 to 55, in 4 racial groups) were reported. There were two participants who were enrolled but did not meet all study inclusion–exclusion criteria, and one participant did not have the injection procedure completed due to a failure of the imaging equipment. Two posterolateral injections using a 22-gauge needle under fluoroscopic guidance delivered Intralink to the mid-coronal plane of each hemi-annulus (left and right) of each symptomatic disc (Figure 3). Each injection contained 48 mM (millimolar) of genipin in a proprietary EPPS-Phosphate buffer with a pH of 9. The volumes of the Intralink reagent injected ranged from 0.5 mL to 1.0 mL based on the cross-sectional dimensions of the target disc observed in pre-treatment MRI scans. Twenty-five lumbar discs were treated in the seventeen participants meeting the study criteria.

The primary endpoints in this study were the occurrence of serious adverse events by 30 days post-procedure and the reduction of patient-reported pain and disability at 3 months. Efficacy endpoints were also assessed at 1–2 weeks, 1-month, 6-month, and 12-month follow-ups. Secondary efficacy endpoints included kinematic parameter changes from baseline at 1- and 3-month follow-ups including segmental flexion–extension instability, changes in the segmental and global lumbar range-of-motion, disc height, and segmental standing lordosis.

This single-arm study of safety and efficacy demonstrated procedure and device safety with the only substantial observation being a temporary and fully treatable inflammatory response beginning 2 to 3 days after leakage of Intralink outside of the disc [35]. Consequently, instructions for use restrict the use of the injectable device to patients with degraded symptomatic discs that are capable of retaining the liquid injectate (i.e., patients with collapsed discs or discs having fissures that penetrate through the outer annulus of the disc should not be treated with this device). Pre-treatment discography may be necessary to screen out patients with penetrating disc fissures. Combined injection and fluoroscopic imaging techniques to minimize the chance of device extravasation were also developed.

**Figure 4:**
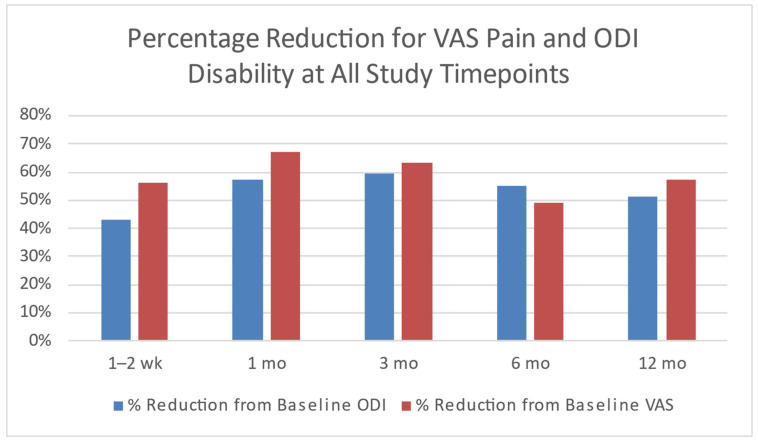
Significant reductions in Visual Analogue Scale (VAS) pain (*p* < 0.001) and Oswestry Disability Index (ODI) disability (*p* < 0.001) compared to baseline at all study timepoints [35].

Clinically meaningful improvements in pain and disability scores occurred in 94% of patients at the 3-month timepoint, with a mean reduction in Visual Analogue Scale (VAS) pain of 59.7% and a mean percentage reduction in Oswestry Disability Index (ODI) disability of 63.4%. Figure 4 shows the improvements in primary patient-reported outcomes at all study timepoints. The percentage of patients with excellent results (with both VAS pain and ODI disability reduced 50% or more from baseline values) or good results (VAS pain decreased 20 mm or more and/or ODI disability decreased 20% or more) was 80% or more from 2 weeks post-treatment to 2 years (only five patients were assessed at 24 months). Intralink treatment was found to be fast-acting with treatment effects seen in all but one of the participants at 1 to 2 weeks post-procedure. This rapid response is in sharp contrast to surgical approaches that typically require a significant period of post-surgical recovery and rehabilitation, and to biological therapies such as platelet-rich-plasma or stem cells which typically require six or more weeks to achieve a desirable effect. In addition, the treatment effects appear to be long-lasting with continued or improved clinical results through 12 months in 80% of patients treated as well as in all five of the patients with 2-year results. In contrast, epidural steroid injections (ESIs) rarely have a treatment effect longer than three months.

**Figure 5:**
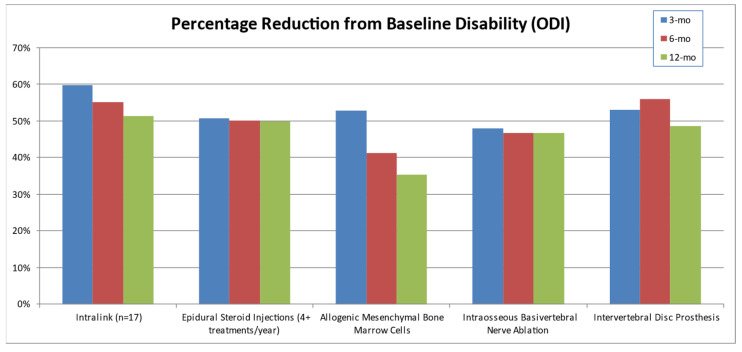
A comparison of Intralink’s Oswestry Disability Index (ODI) reduction [35] with those of conventional and emerging treatments for discogenic low back pain: epidural steroid injections [54], the use of allogenic mesenchymal stem cells [55], intraosseous nerve ablation [56], and lumbar disc prosthesis [57]. Alternative treatment results are from randomized controlled trials with representative or superior efficacy in their class of treatment.

A comparison of ODI disability reductions between Intralink [35] and other conventional and emerging treatments for discogenic low back pain demonstrated similar or superior results for Intralink (Figure 5). The comparative treatment data are from randomized controlled trials with representative or superior efficacy in their class of treatment: epidural steroid injections (four treatments per year) [54], the use of allogenic mesenchymal bone marrow cells [55], intraosseous basivertebral nerve ablation [56], and total lumbar disc replacement (ProDisc II) [57]. In view of this aggregated data, a mean reduction in ODI disability approaching 50% from the pre-treatment baseline represents a favorable clinical outcome for this disorder. The Intralink mean disability reductions exceeded this level of improvement from 1 month (*n* = 17) through 2 years (*n* = 5).

**Figure 6:**
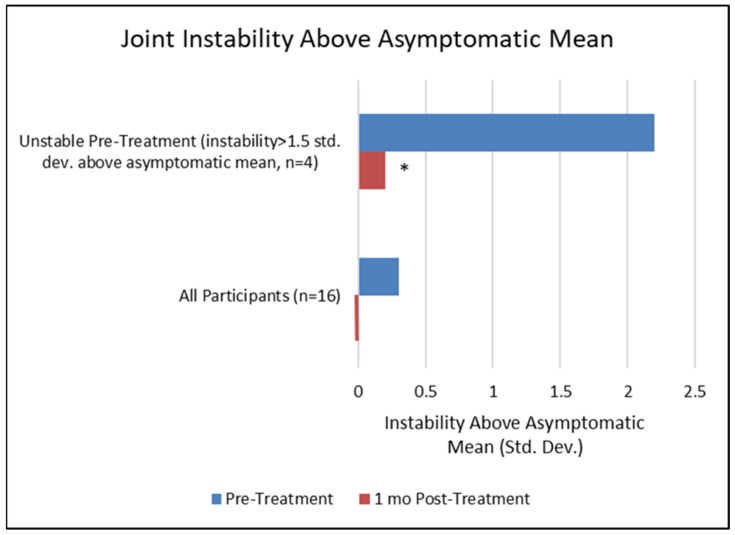
The mean joint instability of Intralink-treated joints for clinically unstable levels and for all scored levels at pre-treatment and 1 month post-treatment (instability score is not calculated when flexion–extension joint motion is less than 3 degrees) [35]. * statistically significant difference, *p* < 0.05.

There was a non-significant trend towards reduced instability for all treated joints at 1 month post-treatment (Figure 6). However, a subset of the symptomatic discs demonstrated clinical instability prior to treatment (instability score was more than 1.5 standard deviations above the mean of an asymptomatic population). Following treatment, the unstable segments had statistically significant instability reductions observed at the 1-month and 3-month follow-ups. The Intralink stabilization brought the instability scores of the clinically unstable joints down to the mean score of an asymptomatic population (difference = 0.2 at 1 month, *p* < 0.05; 0.0 at 3 months, *p* < 0.05), confirming that the joint stabilization capabilities demonstrated in several ex vivo experiments [33,41,42,43,44] can be replicated in vivo, as expected. The corresponding reductions in VAS pain and ODI disability for these four patients were 77% and 75%, respectively. These results also confirm that joint stabilization is an appropriate mechanism to address the immediate clinical need, and that the mechanical stabilization provided by this intra-annular mesh of polymers is sufficient to affect a reduction in discogenic pain and disability. Currently, there is no clinical evidence to confirm the adequacy of Intralink load-sharing and joint stabilization in effectively resisting the otherwise ongoing mechanical degradation of these tissues.

Intralink is not currently approved for commercial use. There are on-going discussions with regulatory agencies to bring the Intralink product to the U.S. and European markets.

### 3.5. The Current Continuum of Care for Discogenic LBP

For well over a century, a primary intent of most non-palliative treatments for progressive disc degeneration and the associated chronic discogenic pain and disability has been to address the mechanical deficiencies of degraded lumbar intervertebral joints—from joint immobilization (fusions) to more recently developed motion constraining devices and disc regenerative technologies. Several factors have limited the success of these mechanical restorative strategies including the morbidity from surgical invasiveness, the inability to restore or replicate the complex spinal joint architecture and mechanics, the harsh biological environment of degenerative discs, and healthcare cost considerations. The need for immediately effective and long-lasting means for stabilizing spinal joints still exists. Innovative solutions should involve low-morbidity and preferably micro-invasive techniques that are not dependent on disc biology to mechanically support and constrain degraded disc tissues. To benefit populations impacted by health care disparities, innovations for the ubiquitous, debilitating, and costly problem of discogenic back pain should be immediately effective to minimize lost work time, long-lasting to avoid return visits and treatments, relatively low-cost, and deliverable in an outpatient clinic or ambulatory surgical center. In sharp contrast, conventional treatment algorithms for chronic or recurrent low back pain patients following the failure of conservative care do not attempt to address the underlying mechanical insufficiency of the disc, resulting in the common sequelae of increasing tissue degradation, pain and disability, along with decades of lost work time and repeated visits to health care professionals. By providing additional mechanical support, Intralink may counteract the mechanically induced aspects of the degenerative process that lead to a cascade of detrimental changes to the disc and adjacent tissues.

**Figure 7:**
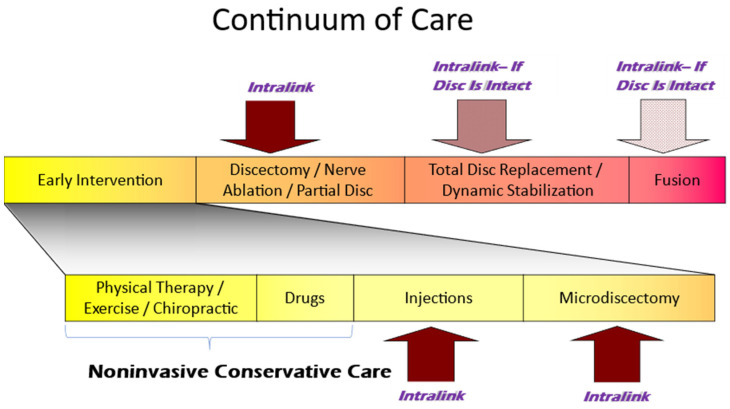
Intralink is indicated for early- to mid-stage care in patients with chronic or recurrent low back pain after the patient has been unresponsive to noninvasive conservative treatment (physical and medical). On the other end of the continuum, Intralink may also be appropriate as a micro-invasive substitute for later stage surgical treatment if disc integrity is confirmed (no full-thickness penetrating fissures).

While this non-biologic nano-tethering approach can effectively reinforce spinal discs and stabilize joints at any stage of disc degeneration, Intralink may be best suited to meet the currently unmet need in early to mid-stage recurrent and chronic LBP patients. Figure 7 shows the placement of the Intralink treatment in the continuum of care for IDD-associated chronic or recurrent low back pain and disability. With earlier stage patients, Intralink intervention may potentially lessen decades of back pain and disability, plus it may decrease the likelihood of disc failures and the resulting radiculopathy such as that with disc herniations. Surgical approaches that do address the progressively decreasing mechanical support are appropriately positioned at the later stages of treatment due to their high costs and complication rates [58], rehabilitation requirements [59], and associated opioid exposure [60,61,62]. Therefore, Intralink presents a unique option in enabling early intervention in resisting disc destabilization that may preclude further, more invasive treatments.

In contrast to Intralink and late-stage IDD surgical approaches, most of the non-surgical, non-invasive, or micro-invasive (injections) treatment options for chronic or repetitive discogenic back pain are palliative in nature, with no possibility of addressing the underlying tissue mechanical deficiencies or altering the natural course of back pain [63]. These palliative treatments are the predominant options for early- to mid-stage disc degeneration cases. Pain-masking approaches may actually accentuate excessive tissue stresses and the resulting tissue degradation by reducing pain-avoidance behaviors.

Of high importance with regard to selecting appropriate candidates for this intervention, knowledge of the pathological processes of symptomatic internal disc disruption and the capability to identify patients with earlier-stage lumbar discogenic pain [64] have advanced considerably during the course of the investigations summarized here. These existing and emerging assessments, outside of the scope of this present review, involve functional MRI, MR spectroscopy and other advanced imaging techniques [64,65,66], the radiographic assessment of sagittal translation, rotations, and instability and associated clinical tests [67,68,69], and provocation or anesthetic discography combined with computed tomography (CT discogram) [64,66].

The traditional course of management for discogenic pain patients for whom conservative medical management approaches are ineffective often involves a treatment gap prior to the use of traditional surgical interventions such as discectomies, fusions, and disc prostheses. The progressive nature of this disorder and its prevalence and societal cost have driven multiple efforts to bridge this gap, attempting to find interventions capable of stemming the ongoing degenerative disorder while providing relief to earlier-stage, recurrent discogenic pain. This review summarizes the reasons why the Intralink technology may be well suited to address this treatment gap, and the gap itself defines the primary clinical indications for this injectable, polymeric medical device. Additional clinical evidence is required to establish the proper utilization of this technology in the broad spectrum of lower back disorders in the midst of a broad scope of existing and emerging interventional treatments for low back pain [70].

## 4. Conclusions

This paper was intended to provide an example of the extensive research journey directed at the development and eventual commercialization of a novel solution for spine care from a concept state to clinical utilization. The paper describes a portion of the evaluation of the clinically relevant capabilities of a new biomaterial technology, and the data-driven determination of its appropriate placement in the clinical care continuum for the treatment of chronic or recurrent discogenic low back pain due to degenerative disc disease.

## Data Availability

No new data were created or analyzed in this study. Data sharing is not applicable to this review article.
